# Posttransplant diffuse anogenital squamous cell carcinomas in situ in an African American woman

**DOI:** 10.1016/j.jdcr.2025.04.018

**Published:** 2025-04-26

**Authors:** Alex Balfour, Olayemi Sokumbi

**Affiliations:** a School of Medicine, University of California, Irvine, Irvine, California; b Department of Dermatology, Mayo Clinic, Jacksonville, Florida

**Keywords:** human papillomavirus, immunosuppressed, skin of color, squamous cell carcinoma in situ, transplant

## Introduction

Squamous cell carcinoma in situ (SCCIS), represents a prevalent form of nonmelanoma skin cancer (NMSC) commonly observed on sun-exposed skin because of the carcinogenesis of UV radiation. SCCIS also manifests in sun-protected areas, including the anogenital region. Risk factors for SCCIS of anogenital skin include human papillomavirus (HPV) infection, and chronic inflammatory dermatoses. Immunosuppression increases the risk of UV and HPV induced SCCIS. In this report, we discuss a case involving an African American woman who, after a solid organ transplant, presented with multiple SCCIS on the groin, vulva, and medial thighs. This case underscores the critical need for vigilant genital skin examinations, particularly in individuals with skin of color who are immunosuppressed.

## Case report

A 50-year-old African American woman presented to our dermatology clinic with numerous black papules on the mons pubis, groin, and medial thighs, which had progressively enlarged over several months. Medical history includes 2 renal transplants, in 2001 and 2012, and chronic immunosuppressive therapy with tacrolimus (0.5 mg twice a day) and prednisone (10 mg every day). In early 2024, an abnormal Pap smear led to the discovery of vaginal intraepithelial neoplasia grade 2. There was no prior history of abnormal Pap smears. Biopsy of the right thigh conducted at an external facility identified SCCIS with follicular extension. One month before presentation, she underwent a series of gynecological procedures including CO_2_ laser treatment of the vagina, excision of a right labial majora lesion, and CO_2_ laser ablation of perianal condylomas that were present before presentation.

**Fig 1:**
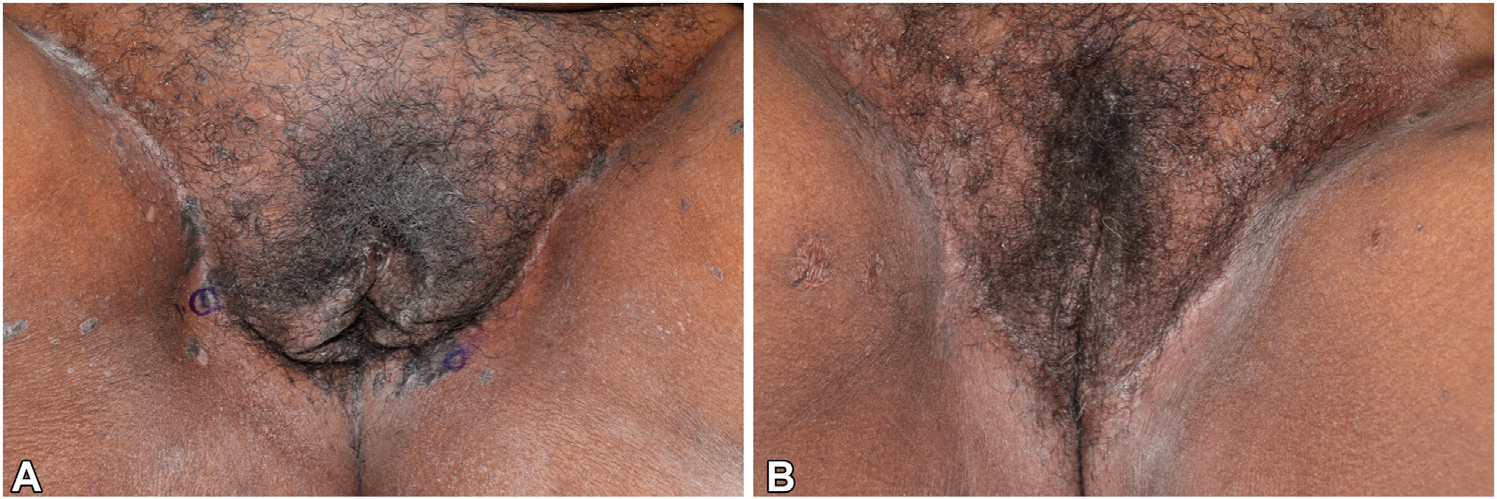
**A,** Involving the mons pubis, groin, labia, perineum, and medial thighs there are 10 to 20 hyperpigmented papules coalescing to forming plaques. **B,** Involving the mons pubis, groin, labia, perineum, and medial thighs there are hyperpigmented patches postoperatively.

**Fig 2:**
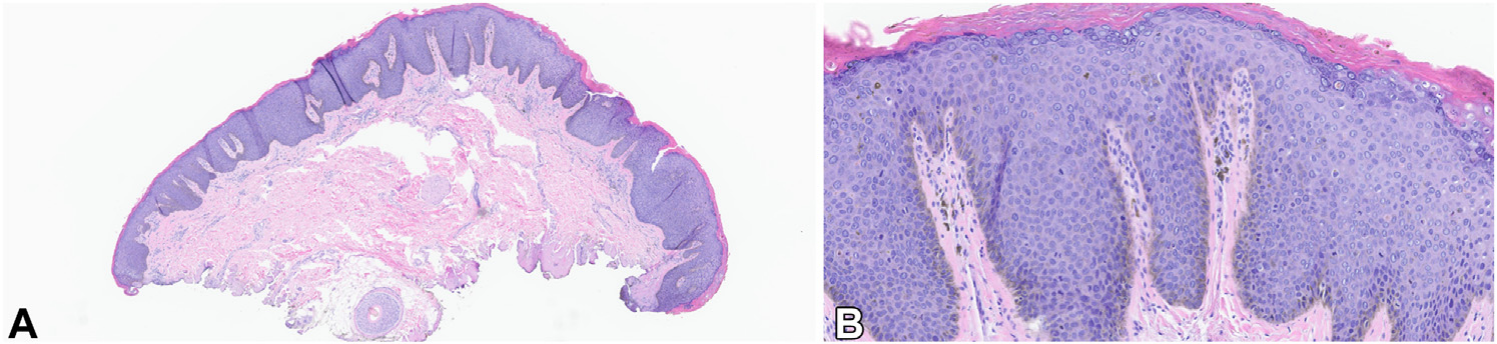
**A,** Shave biopsy with full-thickness cytologic atypia occurring in the background of human papillomavirus-induced cytopathic changes. **B,** Full-thickness atypia highlighting cells with high nuclear/cytoplasmic ratio, nuclear pleomorphism, and scattered mitotic figures. (**A** and **B,** Hematoxylin-eosin stain; original magnifications: **A,** ×5; **B,** ×25.)

A comprehensive skin examination revealed 10 to 20 black, well-demarcated, thin plaques on the suprapubic groin, inguinal crease, and medial thighs, measuring 1 to 2 cm in size (Fig 1, *A*). These plaques lacked a discernible pigment pattern on dermatoscopy. Histopathology of a plaque on the right inguinal crease demonstrated SCCIS/high-grade squamous intraepithelial lesion (Fig 2). Based on recommendations from the American Society of Dermatopathology Appropriate Use Criteria, HPV testing was not performed.^1^

In the operating room, gynecology performed extensive CO_2_ laser eradication of all lesions involving the vulva and mons pubis. Anoscopy revealed small plaques that were excised then fulgurated with electrocautery by colorectal surgery.

Two weeks postoperatively, the patient reports overall well-being with no vulvar or anorectal symptoms. At 4- and 7-month follow-ups she was noted to have postinflammatory inflammation without other adverse effects or recurrence (Fig 1, *B*).

In the setting of a second organ rejection, and increased malignancy risk, she has been tapered off immunosuppression while awaiting a third graft. She will be administered the 3-dose-series of the HPV vaccine while off immunosuppression.

## Discussion

Renal transplant recipients exhibit a 3- to 5-fold increased risk of cancer relative to the general population, with approximately 40% of malignancies being NMSC.^2^ Nonrenal solid organ transplant recipients (SOTR) are also at increased risk of cutaneous squamous cell carcinoma, with thoracic organ recipients at especially increased risk, likely due to need for increased immunosuppression.^3^ Regular comprehensive skin examinations in this population is highly recommended and it is paramount to counsel these patients on their increased risk of NMSCs. Patients with darker skin types (Fitzpatrick skin types III-VI) in particular may not think of themselves as a higher risk population given melanin’s protective effect against UV radiation.

HPV-positive SCCIS, in sun-protected areas such as genital skin, is the most common NMSC in SOTR with darker skin.^4^ Although not all SCCIS requires HPV infection to develop, some types, particularly in the anogenital region, may be induced by it. In contrast, high-grade squamous intraepithelial lesion, a precursor to SCCIS, is full-thickness keratinocyte atypia typically associated with HPV infection. HPV subtypes 16, 18, 31, and 33 are likely to be implicated in SCCIS of sun-protected areas. Subtype testing is rarely appropriate for immunosuppressed adults with SCCIS of the genital skin as it does not change management.^1^

This case of extensive SCCIS in an African American SOTR underscores the necessity of comprehensive skin and genital examinations for patients with skin of color, particularly when they are immunosuppressed. The subtlety of SCCIS in darker skin may complicate early detection. Clinicians should have a low threshold to refer for skin cancer screening in patients that are immunosuppressed. Challenges to management include extensive disease burden, patient comorbidities, coordination of multidisciplinary care, and social determinants of health. Patients may not be in a high resource region with access to a dermatologist or multidisciplinary team, which may delay time to diagnosis and treatment. Symptoms in the anogenital region may be embarrassing to the patient and should be brought up sensitively and empathetically as part of a comprehensive history and physical examination. Education with examples of SCCIS on skin of color would be beneficial to SOTRs of darker skin to empower the patient to bring up new or concerning growths.

For widespread SCCIS, therapeutic strategies such as CO_2_ laser combined with 5-fluorouracil cream or CO_2_ laser plus photodynamic therapy have demonstrated superior outcomes compared with CO_2_ laser monotherapy.^5^^,^^6^ 5-Fluorouracil cream was not used in this patient because of the challenge with application. Other treatment options to consider include Mohs micrographic surgery and wide local excision for less widespread lesions. Modification of immunosuppression is essential to management. If immunosuppression cannot be lowered or tapered off, it may be reasonable to consider switching to an mTOR inhibitor such as sirolimus to potentially reduce NMSC development.^3^

We recommend HPV-vaccination in SOTRs as HPV DNA has been found in 80% of NMSCs in immunosuppressed patients.^3^ One study found that the 9-valent HPV vaccine induced a robust immune response in SOTRs. Although the response was weaker than in healthy individuals, the vaccine is efficacious in preventing HPV-related cancers in SOTR.^7^ Close follow-up is recommended, with skin examinations every 3 months and in a high-risk skin cancer clinic if available. Darker skin types are generally perceived as being low risk of SCCIS; this case illustrates the importance of integrating immunosuppression status into the NMSC risk assessment in patients with skin of color and highlights the need for comprehensive skin and genital examinations for SOTRs. One limitation of this report is the lack of longer follow-up time. Although currently without recurrence, the risk of recurrence still exists especially within the next 1 to 5 years.

## Conflicts of interest

None disclosed.

## References

[1] Vidal C.I., Armbrect E.A., Andea A.A. (2018). Appropriate use criteria in dermatopathology: initial recommendations from the American Society of Dermatopathology. J Cutan Pathol.

[2] Pradita R.A., Adisasmito A.S., Indriatmi W. (2023). Risk of non-melanoma skin cancer in kidney transplantation recipient: an evidence-based case report. Acta Med Indones.

[3] Berman H., Shimshak S., Reimer D. (2022). Skin cancer in solid organ transplant recipients: a review for the nondermatologist. Mayo Clin Proc.

[4] Chung C.L., Nadhan K.S., Shaver C.M. (2017). Comparison of posttransplant dermatologic diseases by race. JAMA Dermatol.

[5] Shen S., Liu X., Yang X., Hu C., Wang P., Wang X. (2022). A combination of laser-assisted ALA-PDT for squamous cell carcinoma in situ and field-directed ALA-PDT for actinic keratosis. Photodiagnosis Photodyn Ther.

[6] Glenn C.J., Parlette E.C., Mitchell C. (2015). Fractionated CO_2_ laser-assisted delivery of topical 5-fluorouracil as a useful modality for treating field cutaneous squamous cell carcinomas. Dermatol Surg.

[7] Boey L., Curinckx A., Roelants M. (2021). Immunogenicity and safety of the 9-valent human papillomavirus vaccine in solid organ transplant recipients and adults infected with human immunodeficiency virus (HIV). Clin Infect Dis.

